# Wogonoside prevents colitis-associated colorectal carcinogenesis and colon cancer progression in inflammation-related microenvironment via inhibiting NF-κB activation through PI3K/Akt pathway

**DOI:** 10.18632/oncotarget.8815

**Published:** 2016-04-18

**Authors:** Yang Sun, Yue Zhao, Xiaoping Wang, Li Zhao, Wenjun Li, Youxiang Ding, Lingyi Kong, Qinglong Guo, Na Lu

**Affiliations:** ^1^ State Key Laboratory of Natural Medicines, Department of Natural Medicinal Chemistry, China Pharmaceutical University, Nanjing 210009, China; ^2^ Jiangsu Key Laboratory of Carcinogenesis and Intervention, China Pharmaceutical University, Nanjing 210009, China; ^3^ Jiangsu Key Laboratory of Drug Design and Optimization, China Pharmaceutical University, Nanjing 210009, China

**Keywords:** wogonoside, AOM/DSS mouse model, colitis-associated cancer, NF-κB

## Abstract

The inflammatory microenvironment has been reported to be correlated with tumor initiation and malignant development. In the previous studies we have found that wogonoside exerts anti-neoplastic and anti-inflammatory activities. In this study, we aimed to further investigate the chemopreventive effects of wogonoside on colitis-associated cancer and delineated the potential mechanisms. In the azoxymethane initiated and dextran sulfate sodium (AOM/DSS) promoted colorectal carcinogenesis mouse model, wogonoside significantly reduced the disease severity, lowered tumor incidence and inhibited the development of colorectal adenomas. Moreover, wogonoside inhibited inflammatory cells infiltration and cancer cell proliferation at tumor site. Furthermore, wogonoside dramatically decreased the secretion and expression of IL-1β, IL-6 and TNF-α as well as the nuclear expression of NF-κB in adenomas and surrounding tissues. *In vitro* results showed that wogonoside suppressed the proliferation of human colon cancer cells in the inflammatory microenvironment. Mechanistically, we found that wogonoside inhibited NF-κB activation via PI3K/Akt pathway. In conclusion, our results demonstrated that wogonoside attenuated colitis-associated tumorigenesis in mice and inhibited the progression of human colon cancer in inflammation-related microenvironment via suppressing NF-κB activation by PI3K/Akt pathway, indicating that wogonoside could be a promising therapeutic agent for colorectal cancer.

## INTRODUCTION

Colorectal cancer (CRC) is the second commonest malignancy in the world with high mortality [1, 2]. However, the precise mechanisms and etiological factors leading to the initiation and development of colon cancer still remain unclear. Increasing evidence has suggested that inflammation is correlated with in tumor initiation and malignant progression [3-7]. Moreover, the close link between chronic inflammation and the development of CRC is supported by epidemiological studies. Therefore, patients with inflammatory bowel disease (IBD) have an increased risk of developing CRC.

The key components of inflammation include primary inflammatory cytokines, hematopoietic growth factors (MCSF), and the master transcription factor NF-κB [8-10]. The inflammatory microenvironment facilitates tumorigenesis through a series of dynamic and reciprocal interactions between inflammatory cells and tumor cells [11]. There are multiple immune cell populations implicated in the crosstalk within the tumor microenvironment [12]. Monocytes, the primary producers of cytokines during inflammation, not only play key roles in the innate immune system but also migrate to hypoxic sites such as growing tumor mass [13]. These cells in the tumor microenvironment promoted tumor initiation, accelerated tumor progression, angiogenesis and metastasis by releasing a variety of growth factors, chemokines and cytokines [11, 14-17].

NF-κB, regarded as primary regulator of inflammatory responses, can promote the initiation and amplification of inflammation [18, 19]. The pro-inflammatory stimuli activate NF-κB, which triggers the transcription of mainly pro-inflammatory and anti-apoptotic target genes at least during an initial phase. Moreover, prolonged or imbalanced NF-κB activation may favor tumorigenesis [18]. NF-κB activation may link inflammation to tumor initiation and promotion by regulating the target genes [18, 20]. Therefore, NF-κB could facilitate the development of colitis associated colorectal cancer by sustaining the ongoing inflammatory process in the gut mucosa.

The phosphatidylinositol-3-kinases (PI3K) are a conserved family of signal transduction enzymes that are correlated with cellular proliferation and survival [21, 22]. More specifically, the PI3Ks and the downstream serine/threonine kinase Akt regulate cellular activation, inflammatory responses, cycle entry, survival and apoptosis [22]. The activation of PI3K/Akt can promote cell survival by indirectly activating the pro-survival transcription factor NF-κB through the phosphorylation of I-κB kinase (IKK) [23, 24].

Wogonoside, a glucuronide metabolite of bioactive flavonoid wogonin, possesses anti-cancer [25] and anti-inflammation-induced angiogenic activities [26]. Recently, our study has demonstrated that wogonoside exerts the protective effect on DSS-induced colitis [27]. In the present study, we further investigated the anti-cancer effect of wogonoside on colitis-associated tumorigenesis and colon cancer cell proliferation in the inflammatory microenvironment. Furthermore, we illuminated the underlying mechanisms and addressed that wogonoside exhibited the anti-cancer effect via inhibiting NF-κB activation through PI3K/Akt pathway.

## RESULTS

### Wogonoside attenuated AOM/DSS-induced colitis-associated tumorigenesis

**Figure 1:**
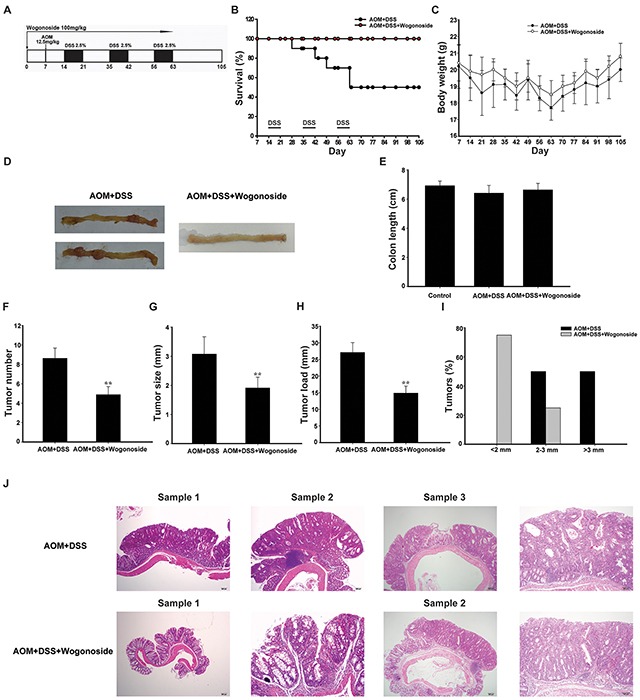
Wogonoside treatment decreased the incidence and development of AOM/DSS-induced CAC in mice **A.** Diagram shows the experimental course of AOM/DSS mouse model. **B.** Kaplan–Meier survival curves show the effect of wogonoside on the survival of AOM/DSS-treated mice. **C.** Basal body weight changes of all groups after AOM/DSS induction of CAC (n= 8 mice per group) **D.** Macroscopic appearances of colon and data statistics of colon length **E.** after AOM/DSS induction of CAC at day 105 (n= 8 mice per group). **F.** Tumor numbers were counted on day 105. **G.** Tumor sizes were determined using Spot software for microscopic tumors or a caliper for macroscopic tumors (n= 8 mice per group). **H.** Average tumor load was determined (n= 8 mice per group). **I.** Histogram showing the size distribution of tumors. **J.** H&E stains of serial sections of colons. Data are presented as mean ± SD. **P < 0.01 compared with AOM/DSS group.

To determine the protective effect of wogonoside on colitis-associated cancer (CAC) development, we used the well-established AOM/DSS model in C57BL/6 mice (Figure 1A). Wogonoside was well tolerated in mice, and no observable toxicity and gross change in any organ examined in all groups up to 105 days (Table 1). Kaplan–Meier survival curves showed that wogonoside treatment dramatically increased the survival of AOM/DSS-treated mice during the experiment (Figure 1B). Bloody stool and body weight loss were observed in AOM/DSS group throughout the study when the mice received 2.5% DSS in drinking water. However, wogonoside regained the body weight of AOM/DSS mice (Figure 1C). As shown in Figure 1C, the mean body weight at the termination (week 15) was not different among the groups.

**Table 1:** Brief Assessment of the Colitis-associated Cancer Model

Groups	Weight (g)		
	Spleen	Liver	Lung
**Normal**	0.092 ± 0.009	1.104 ± 0.089	0.142 ± 0.009
**AOM/DSS**	0.101 ± 0.012	1.129 ± 0.106	0.167 ± 0.015
**AOM/DSS+wogonoside**	0.095 ± 0.010	1.110 ± 0.105	0.156 ± 0.024

Each data point represents the mean ± SD of six animals for each group.

After the mice were sacrificed, nodular polypoid tumors were observed in the middle and distal colon of all mice in the AOM/DSS group. A few nodular tumors were found in the distal colon in wogonoside-treated group (Figure 1D). As shown in Figure 1E, the mean colon length of AOM/DSS group was slightly shorter than that of wogonoside treatment group without statistically significant at day 105. The assessment of tumor number, tumor size and tumor load showed that wogonoside decreased tumor number, tumor size and average tumor load in AOM/DSS model (Figure 1F-1H). Moreover, we found a lower frequency of large sized adenomas in wogonoside-treated group than in AOM/DSS group (Figure 1I).

Mucosal inflammation, aberrant crypt foci adenoma, ulcer, or dysplasia was found in the colon tissues in model groups by histological examination. As shown in Figure 1J, the results of hematoxylin and eosin (H&E) staining showed that samples in AOM/DSS group had a large adenocarcinoma inside mucosa with several abnormal cells exhibiting cylindrical shape, large nuclei, increasing nuclear/cytoplasmic (N/C) ratio and cellular cleavage. Conversely, wogonoside remarkably relieved the severe condition. Taken together, these results suggested that wogonoside inhibited inflammation-associated carcinogenesis and tumor development in the AOM/DSS CAC model.

### Wogonoside diminished AOM/DSS-induced colonic massive infiltration of inflammatory cells and inhibited the development of colitis-associated colon cancer

**Figure 2:**
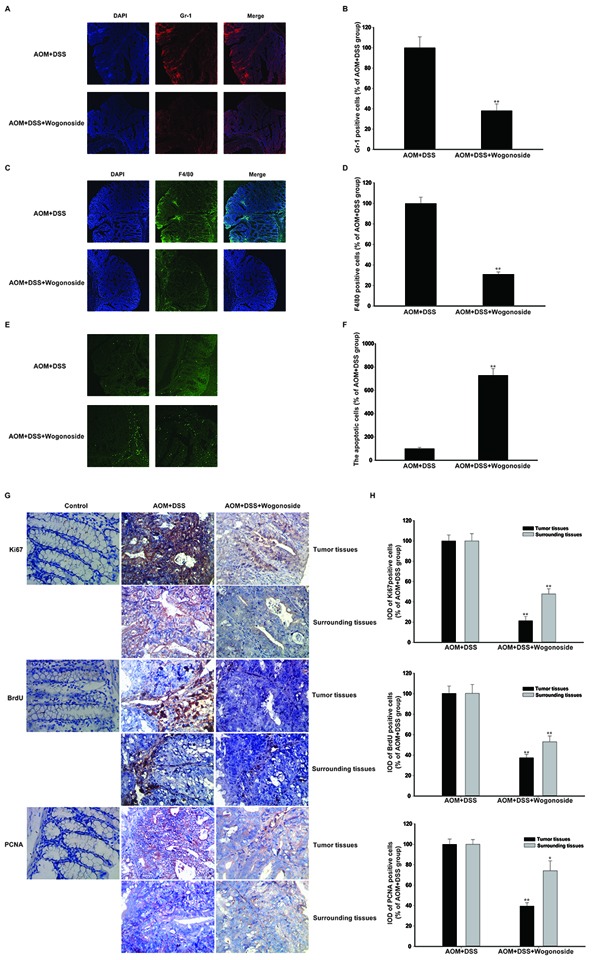
Wogonoside inhibited inflammatory cells infiltration and colon cancer progression in the tumor inflammatory microenvironment *in vivo* **A–B.** The distribution of neutrophil (Gr-1^+^) and **C–D.** macrophage (F4/80^+^) infiltration were observed by confocal laser-scanning microscope. The results are representative of three independent experiments and expressed as mean ± SD, **P < 0.01 compared with AOM/DSS group. The results are representative of three independent experiments and expressed as mean ± SD, **P < 0.01 compared with AOM/DSS group. **E–F.** Immunofluorescent TUNEL staining was detected. **G–H.** Immunohistochemistry of Ki-67, BrdU and PCNA in surrounding and tumor tissues were shown with brown colored positive cells. The results are representative of three independent experiments and expressed as mean ± SD, *P < 0.05, **P < 0.01 compared with AOM/DSS group.

The state of chronic inflammation leads to enhanced cell proliferation, cell survival, and tumorigenesis, thus is believed to be directly responsible for the neoplastic transformation of the overlying intestinal epithelium [5, 28]. To assess the severity and involvement of inflammation in the colons of AOM/DSS-treated mice, we stained colon tissue sections to detect neutrophil (Gr-1^+^) and macrophage (F4/80^+^) infiltration [29]. More intriguingly, a higher neutrophils and macrophages accumulation was observed in tumors of mice treated with AOM/DSS. In contrast, wogonoside significantly reduced the recruitment of these inflammatory cells into the tumor site (Figure 2A-2D). Accordingly, we assessed variations in cancer cell proliferation and apoptosis in colonic adenocarcinomas between the wogonoside-treated and AOM/DSS groups. TdT-mediated dUTP-biotin nick end labeling (TUNEL) staining showed that wogonoside increased the apoptosis compared with the AOM/DSS group (Figure 2E and 2F). Ki-67, BrdU and proliferating cell nuclear antigen (PCNA) immunohistochemistry staining showed a substantial reduction of Ki-67, BrdU- and PCNA-labeled cells in wogonoside-treated mice than those of AOM/DSS-treated mice (Figure 2G and 2H). Altogether, our data indicated that wogonoside inhibited AOM/DSS-induced colonic massive infiltration of inflammatory cells and the development of colitis-associated colon cancer.

### Wogonoside reduced production of pro-inflammatory cytokines in colon of AOM/DSS CAC mice and inhibited NF-κB activation via regulating PI3K/Akt pathway

**Figure 3:**
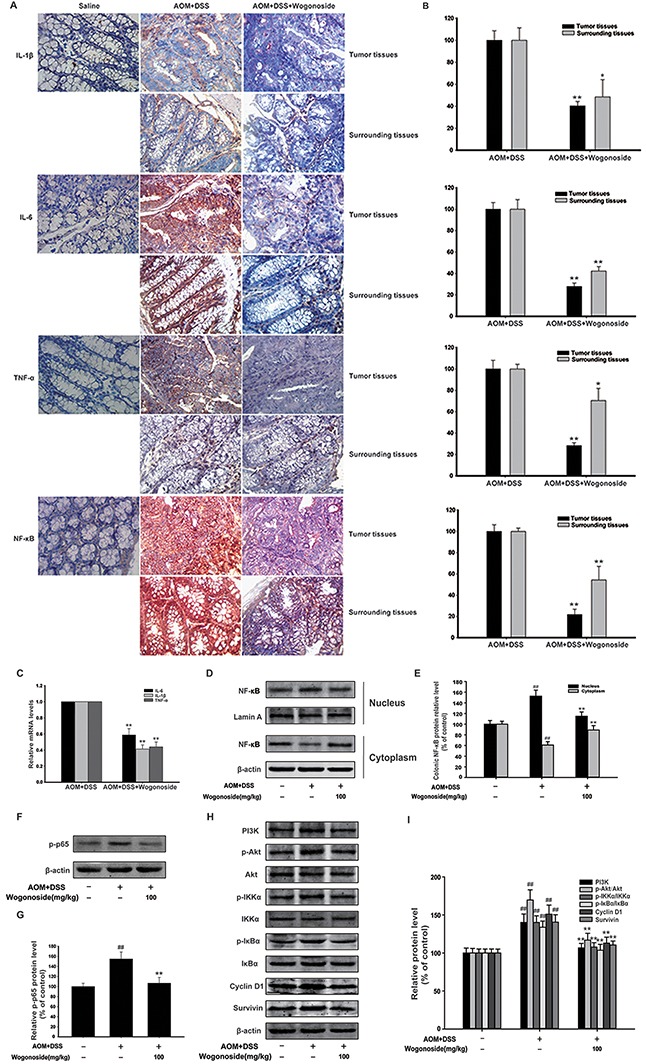
Wogonoside reduced production of pro-inflammatory cytokines and inhibited NF-κB activation via regulating PI3K/Akt pathway in AOM/DSS-induced adenocarcinoma **A–B.** The expression of IL-1β, IL-6, TNF-α and NF-κB p65 in surrounding and tumor tissues of AOM/DSS-treated mice was performed by immunohistochemistry. The results are representative of three independent experiments and expressed as mean ± SD, *P < 0.05, **P < 0.01 compared with AOM/DSS group. **C.** The mRNA levels of IL-1β, IL-6 and TNF-α in tumor tissues. The results are representative of three independent experiments and expressed as mean ± SD, **P < 0.01 compared with AOM/DSS group. **D.** NF-kB p65 nuclear translocation in tumor tissues were determined by Western Blot. **E.** Densitometric analysis was performed to determine the relative ratios of each protein. Lamin A and β-actin were used as nuclear and cytoplasmic markers, respectively. The results are representative of three independent experiments and expressed as mean ± SD, ## P<0.01 compared with normal group; **P<0.01 compared with AOM/DSS group. **F.** The protein expression of p-p65 in tumor tissues. **G.** Densitometric analysis to determine the relative ratio normalized to β-actin. The results are representative of three independent experiments and expressed as mean ± SD, ## P<0.01 compared with normal group; **P<0.01compared with AOM/DSS group. **H.** The protein expression of PI3K, p-Akt, Akt, p-IKKα, IKKα, p-IκBα, IκBα, Cyclin D1 and survivin in tumor tissues was analyzed by Western Blot. **I.** Densitometric analysis to determine the relative ratio normalized to β-actin. The results are representative of three independent experiments and expressed as mean ± SD, ## P<0.01 compared with normal group; **P<0.01compared with AOM/DSS group.

A number of studies have suggested that pro-inflammatory cytokines are associated with inflammation-associated cancer [30-32]. We found that IL-1β, IL-6 and TNF-α were expressed at relatively high levels in AOM/DSS model; however, wogonoside effectively suppressed the expression of IL-1β, IL-6 and TNF-α (Figure 3A and 3B). Moreover, wogonoside inhibited the mRNA levels of IL-1β, IL-6 and TNF-α in tumor tissues (Figure 3C). Our results showed that wogonoside inhibited nuclear NF-κB p65 protein expression in tumor tissues of AOM/DSS treated mice (Figure 3A and 3B, Figure 3D-3E). We also found that wogonoside inhibited the protein expression of p-p65 in tumor tissues of AOM/DSS treated mice (Figure 3F and 3G). As we all know, the PI3K/Akt pathway is important in colon cancer and the activation of PI3K/Akt can activate the downstream target NF-κB through the phosphorylation of IKK [23]. In the present study, we found that wogonoside suppressed PI3K, p-Akt protein expression without affecting the total Akt protein level and inhibited phosphorylation of IKKα and IκBα (Figure 3H and 3I). Moreover, wogonoside decreased the protein expression of Cyclin D1 and survivin, both of which are associated with increased tumorigenesis (Figure 3H and 3I). All above results indicated that wogonoside could inhibit NF-κB activation through PI3K/Akt pathway to alleviate the inflammation-induced injury, and prevented the incidence and the development of CAC.

### Wogonoside inhibited the growth of human colon cancer cells exposed to the conditioned media from LPS-activated THP-1 cells

**Figure 4:**
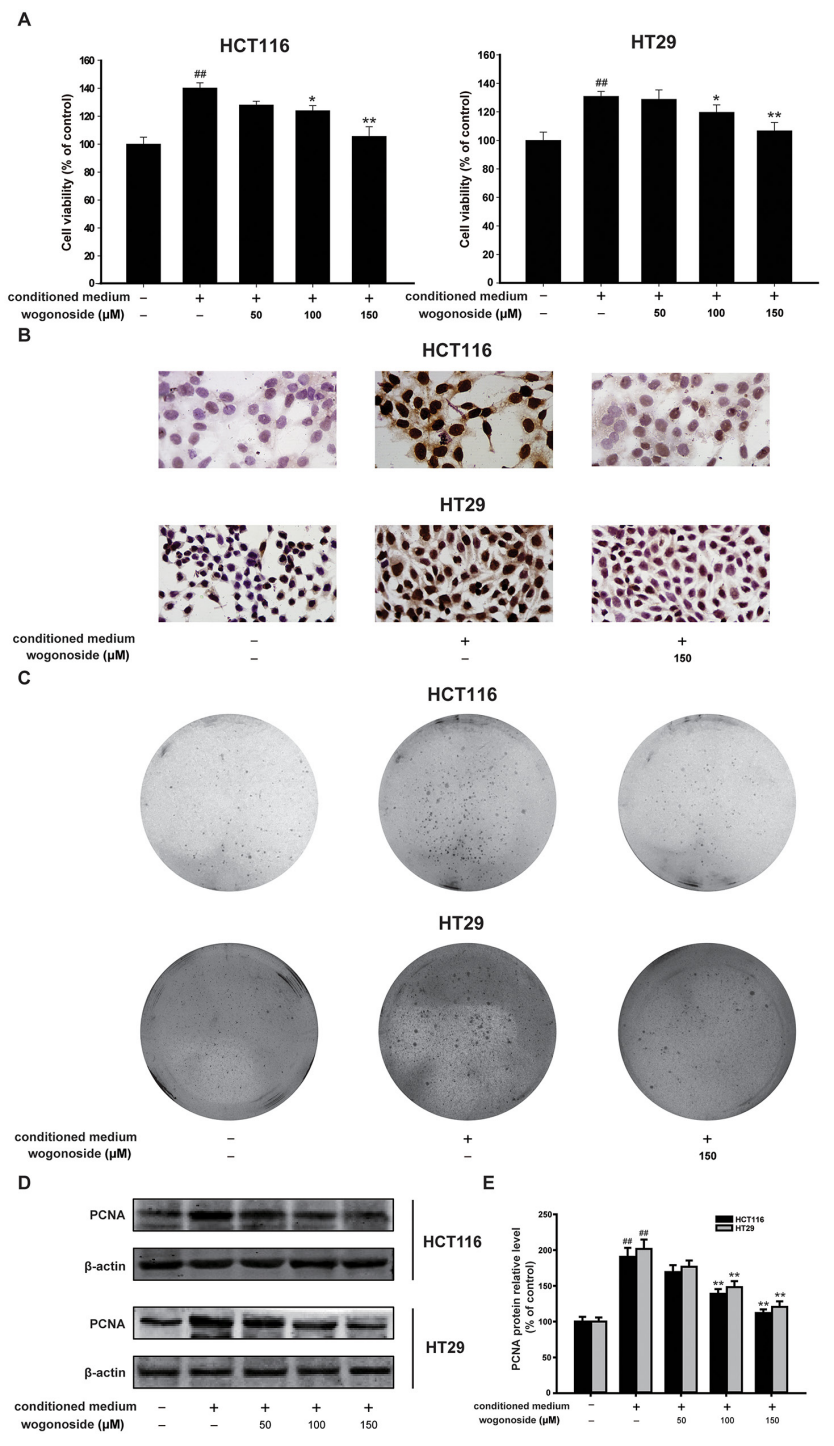
Wogonoside inhibited the growth of human colon cancer cells cultured with the conditioned media from LPS-activated THP-1 cells **A.** MTT assays of HCT-116 and HT29 cells were performed to detect the inhibitory effect of wogonoside on the cell viability. The results are representative of three independent experiments and expressed as mean ± SD, ## P<0.01 compared with control group; *P<0.05, **P<0.01 compared with conditioned media group. **B.** Ki67 cell proliferation detection of HCT-116 and HT29 cells in conditional culture system treated with wogonoside. **C.** Soft-sugar-colony forming experiment was performed to ascertain the inhibitory effect of wogonoside on cell proliferation. **D.** The protein expression of PCNA was analyzed by Western Blot. **E.** Densitometric analysis to determine the relative ratio normalized to β-actin. The results are representative of three independent experiments and expressed as mean ± SD, ## P<0.01 compared with control group; **P<0.01 compared with conditioned media group.

In the current study, we used the conditioned-culture system, in which HCT116 and HT29 colorectal carcinoma cells were exposed to the conditional media from lipopolysaccharide (LPS)-stimulated THP-1 cells, to investigate the effect of wogonoside on inflammation-related cancer progression. We found that the conditional media from LPS-induced THP-1 cells promoted the growth of HCT116 and HT29 colorectal carcinoma cells (Figure 4A). However, wogonoside treatment markedly inhibited the cell growth in HCT116 and HT29 cells supported by the conditional medium in a concentration-dependent manner (Figure 4A). Ki67 assay showed that wogonoside inhibited the proliferation of HCT116 and HT29 cells in the conditional culture system (Figure 4B). The soft-sugar-colony forming experiment was also performed to ascertain the inhibitory effect of wogonoside on cellular proliferation of HCT116 and HT29 cells in the conditional medium (Figure 4C). Furthermore, PCNA protein expression was raised in HCT116 and HT29 cells supported by the conditional medium. On the contrary, wogonoside treatment reversed the change (Figure 4D and 4E). These findings suggested that wogonoside suppressed the proliferation of HCT116 and HT29 cells induced by conditional medium from LPS-stimulated THP-1 cells.

### Wogonoside inhibited the activation of NF-κB pathway induced by the conditioned media from LPS-activated THP-1 cells in human colon cancer cells

**Figure 5:**
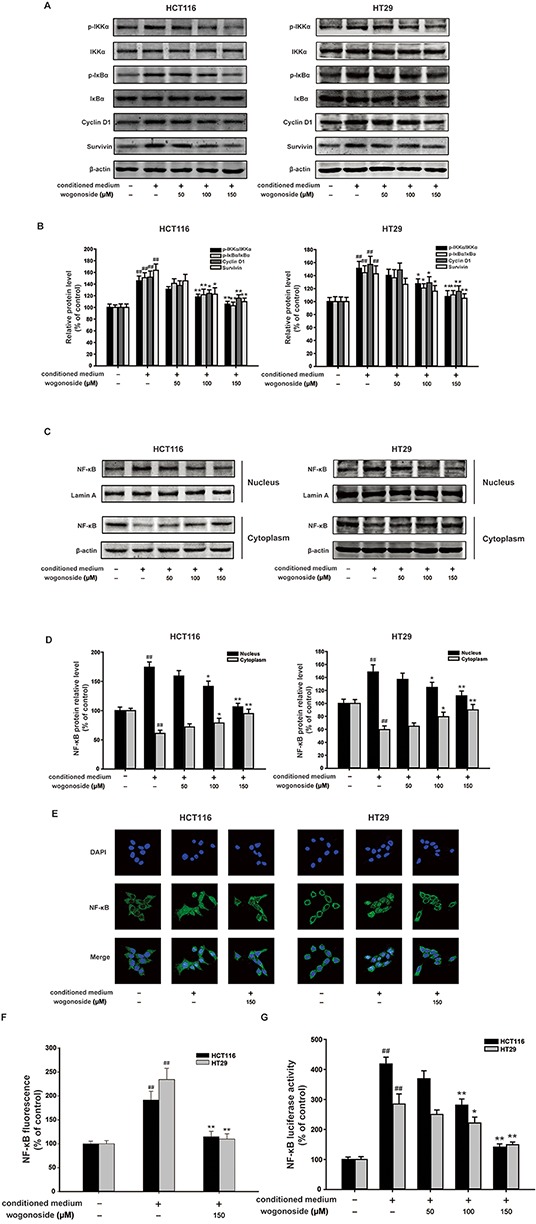
Wogonoside inhibited the activation of NF-κB induced by the conditioned media from LPS-activated THP-1 cells in human colon cancer cells **A.** The protein expression of p-IKKα, IKKα, p-IκBα, IκBα, Cyclin D1 and survivin in HCT116 and HT29 cells in all groups was analyzed by Western Blot. **B.** Densitometric analysis to determine the relative ratio normalized to β-actin. The results are representative of three independent experiments and expressed as mean ± SD. ##P < 0.01 compared with control group; *P < 0.05, **P < 0.01 compared with conditioned media group. **C.** NF-κB p65 nuclear translocation in HCT116 and HT29 cells in all group were determined by Western Blot. **D.** Densitometric analysis was performed to determine the relative ratios of each protein. Lamin A and β-actin were used as nuclear and cytoplasmic markers, respectively. The results are representative of three independent experiments and expressed as mean ± SD. ##P < 0.01 compared with control group; *P < 0.05, **P < 0.01 compared with conditioned media group. **E–F.** Immunofluorescence was performed to analyze NF-κB p65 nuclear translocation in HCT116 and HT29 cells. The results are representative of three independent experiments and expressed as mean ± SD. ##P < 0.01 compared with control group; **P < 0.01 compared with conditioned media group. **G.** The transcriptional activities of p-NF-κB p65 in HCT116 and HT29 cells were determined by Luciferase activity assay. The results are representative of three independent experiments and expressed as mean ± SD. ##P < 0.01 compared with control group; *P < 0.05, **P < 0.01 compared with conditioned media group.

Dysregulation of NF-κB pathway is correlated with the cancer development [10, 33, 34]. Our data showed that wogonoside markedly inhibited phosphorylation of IKKα and IκBα in HCT116 and HT29 cells compared to the conditional medium group (Figure 5A and 5B). Western blot analysis and immunofluorescence staining demonstrated that wogonoside markedly suppressed conditional medium-induced NF-κB nuclear translocation (Figure 5C-5F). Moreover, the Luciferase reporter gene assay revealed that wogonoside decreased transcriptional activity of NF-κB induced by conditional medium in a concentration-dependent manner (Figure 5G). As shown in Figure 5A and 5B, the conditional medium enhanced the protein expression of NF-κB downstream targets Cyclin D1 and survivin, whereas wogonoside reduced the protein expression. These results elucidated that wogonoside inhibited the activation of NF-κB pathway induced by the conditioned media from LPS-activated THP-1 cells in human colon cancer cells.

### Wogonoside inhibited the proliferation of human colon cancer cells in the conditioned media from LPS-activated THP-1 cells via inhibition of NF-κB activation through PI3K/Akt pathway

**Figure 6:**
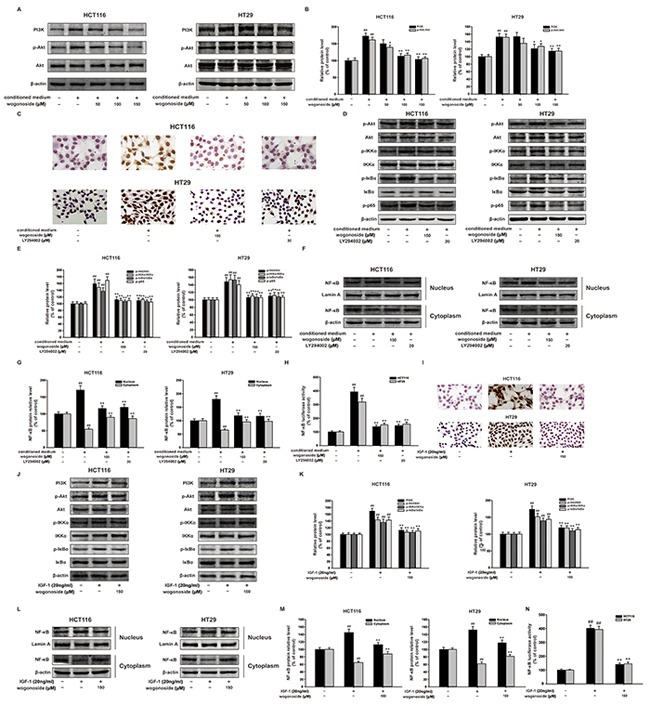
Wogonoside inhibited the proliferation of human colon cancer cells exposed to the conditioned media from LPS-activated THP-1 cells via inhibition of NF-κB activation through PI3K/Akt pathway **A.** The protein expression of PI3K, p-Akt, Akt in HCT116 and HT29 cells in all groups was analyzed by Western Blot. **B.** Densitometric analysis to determine the relative ratio normalized to β-actin. The results are representative of three independent experiments and expressed as mean ± SD. ##P < 0.01 compared with control group; *P < 0.05, **P < 0.01 compared with conditioned media group. (C–H) HCT116 and HT29 cells were cultured in the conditional culture system with wogonoside (150 μM) or LY294002 (20 μM) for 24 h. **C.** Ki67 cell proliferation detection of HCT-116 and HT29 cells in the conditional culture system treated with wogonoside or LY294002. **D.** The protein expression of p-Akt, Akt, p-IKKα, IKKα, p-IκBα, IκBα and p-p65 was analyzed by Western Blot. **E.** Densitometric analysis to determine the relative ratio normalized to β-actin. The results are representative of three independent experiments and expressed as mean ± SD, ## P<0.01 compared with control group; **P<0.01 compared with conditioned media group. **F.** NF-κB p65 nuclear translocation in HCT116 and HT29 cells in all group were determined by Western Blot. **G.** Densitometric analysis was performed to determine the relative ratios of each protein. Lamin A and β-actin were used as nuclear and cytoplasmic markers, respectively. The results are representative of three independent experiments and expressed as mean ± SD. ##P < 0.01 compared with control group; **P < 0.01 compared with conditioned media group. **H.** The transcriptional activities of p-NF-κB p65 in HCT116 and HT29 cells in all groups were determined by Luciferase activity assay. The results are representative of three independent experiments and expressed as mean ± SD. ##P < 0.01 compared with control group; *P < 0.05, **P < 0.01 compared with conditioned media group. (I–M) HCT116 and HT29 cells were treated with IGF-1 (20 ng/ml) with or without wogonoside with for 24 h. **I.** Ki67 cell proliferation detection of HCT-116 and HT29 cells treated with IGF-1 with or without wogonoside. **J.** The protein expression of PI3K, p-Akt, Akt, p-IKKα, IKKα, p-IκBα and IκBα was analyzed by Western Blot. **K.** Densitometric analysis to determine the relative ratio normalized to β-actin. The results are representative of three independent experiments and expressed as mean ± SD, ## P<0.01 compared with control group; **P<0.01 compared with IGF-1 group. L. NF-κB p65 nuclear translocation in HCT116 and HT29 cells in all groups were determined by Western Blot. **M.** Densitometric analysis was performed to determine the relative ratios of each protein. Lamin A and β-actin were used as nuclear and cytoplasmic markers, respectively. The results are representative of three independent experiments and expressed as mean ± SD. ##P < 0.01 compared with control group; **P < 0.01 with IGF-1 group. N. The transcriptional activities of p-NF-κB p65 induced by IGF-1 in HCT116 and HT29 cells were determined by Luciferase activity assay. The results are representative of three independent experiments and expressed as mean ± SD. ##P < 0.01 compared with control group; **P < 0.01 with IGF-1 group.

It has been well documented that NF-κB is the down-stream of PI3K/Akt pathway. Our results showed that wogonoside reduced the protein expression of PI3K and p-Akt with constant total Akt protein level, which indicated the inhibitory effect of wogonoside on PI3K/Akt pathway (Figure 6A and 6B).

Specific PI3K inhibitor LY294002 was used to testify the inhibitory effect of wogonoside on cell proliferation via PI3K/Akt and NF-κB pathways. Interestingly, Ki67 cell proliferation detection showed that LY294002 and wogonoside decreased the proportions of Ki67 positive cells in total cells compared with the conditional medium group (Figure 6C). Moreover, LY294002 and wogonoside could both inhibit the phosphorylation of Akt, IKKα, IκBα and p65 in HCT116 and HT29 cells compared to conditional medium group (Figure 6D and 6E). In addition, LY294002 and wogonoside strikingly suppressed conditional medium-induced nuclear translocation and transcriptional activity of NF-κB (Figure 6F-6H).

To further prove the molecular mechanism, IGF-1, an activator of PI3K/Akt pathway was used to verify the inhibitory effect of wogonoside. Not surprisingly, we found that wogonoside inhibited IGF-1-induced cell proliferation (Figure 6I). Wogonoside decreased the protein expression of p-Akt, p-IKKα and p-IκBα in HCT116 and HT29 cells compared to IGF-1 group (Figure 6J and 6K). Furthermore, wogonoside suppressed IGF-1-induced NF-κB nuclear translocation (Figure 6L and 6M). Wogonoside also inhibited IGF-1-induced transcriptional activity of NF-κB (Figure 6N). All of these results suggested that wogonoside might inhibit the proliferation of human colon cancer cells in the conditioned media from LPS-activated THP-1 cells via suppressing NF-κB activation through PI3K/Akt pathway.

## DISCUSSION

The global incidence rates of colorectal cancer have been continuously increasing in the past years. Chemotherapeutic regimens, including cisplatin, are routinely used in the treatment of advanced-stage colon cancer with poor response rate, apparent side effects and toxicity [35, 36]. Therefore, it is urgent to identify novel chemopreventive agents with minimal or no side effects and toxicities.

Wogonoside is a glucuronide metabolite of bioactive flavonoid wogonin which has anti-inflammatory, antiviral, anti-oxidant, and anti-cancer effects [37]. Our previous study has reported that wogonoside exerts protective effect on DSS-induced colitis [27]. In this study, we investigated the anti-cancer effect of wogonoside on colitis-associated tumorigenesis and colon cancer cell proliferation in inflammatory microenvironment.

Inflammatory bowel disease (IBD) is a well-known example of the tight link between inflammation and cancer [38]. In the present study we used the well-established AOM/DSS model, which is conveniently and widely applied to the study of the prevention of colon cancer. Our results demonstrated that wogonoside dramatically increased the survival of AOM/DSS-treated mice (Figure 1B). Wogonoside also decreased tumor number, tumor size, average tumor load and occurrence of large sized adenomas in the AOM/DSS CAC model (Figure 1F-1I). Moreover, wogonoside remarkably relieved the severe pathological condition (Figure 1J). These findings suggested that wogonoside inhibited inflammation-associated carcinogenesis and cancer development in the AOM/DSS CAC model.

The common pathological changes related to colitis-associated and sporadic CRC involve a tumor microenvironment established by various types of dysregulated immune cells and endothelial cells [15, 39]. The recent studies have suggested that the model of cancer formation comprising six cancer traits proposed by Hanahan-Weinberg [40] has to be updated, and that a seventh character should be immunity and a cancer promoting pro-inflammatory microenvironment [41, 42]. Tumors would become addicted to proliferative and survival signals from the abnormal tumor microenvironment [43]. Our results showed that wogonoside significantly inhibited the neutrophil (Gr-1^+^) and macrophage (F4/80^+^) infiltration into tumor tissues (Figure 2A and 2B). We also found a substantial reduction of Ki-67, BrdU- and PCNA-labeled cells in wogonoside-treated mice than those of AOM/DSS-treated mice (Figure 2D). In addition, wogonoside effectively decreased the protein expression of pro-inflammatory cytokines IL-1β and IL-6 (Figure 3A). Our data indicated that wogonoside inhibited AOM/DSS-induced colonic massive infiltration of inflammatory cells and prevented the development of colitis-associated colon cancer.

A tumor can achieve enhanced NF-κB activity through increased cytokine release from the tumor microenvironment characterized by a chronic inflammatory condition [44, 45, 46]. Furthermore, NF-κB signaling controls a great many of other well conserved cellular processes, including cell proliferation [33, 34]. The PI3K/Akt signaling pathway can promote cell survival by indirectly activating NF-κB through the phosphorylation of I-κB kinase (IKK) [23, 24]. We further determined the mechanisms of the inhibitory effect of wogonoside on colitis-associated colon cancer. The results demonstrated that wogonoside inhibited NF-κB activation through PI3K/Akt pathway induced by AOM/DSS *in vivo* (Figure 3). To confirm our conclusion, we established the conditioned-culture system (human colon cancer cells exposed to the conditioned media from LPS-activated THP-1 cells) *in vitro*. The MTT assay, Ki67 detection, soft-sugar-colony forming experiment and Western blot analysis suggested that wogonoside suppressed the proliferation of HCT116 and HT29 cells in the conditional culture system (Figure 4). Moreover, wogonoside decreased the phosphorylation of IKKα and IκBα, inhibited the nuclear translocation and transcriptional activity of NF-κB of HCT116 and HT29 cells in the conditional culture system (Figure 5). Furthermore, our findings demonstrated that wogonoside inhibited the proliferation of human colon cancer cells in the conditional culture system via suppressing NF-κB activation through PI3K/Akt pathway (Figure 6). All data indicated that wogonoside inhibited the proliferation of human colon cancer cells in the conditioned-culture system via NF-κB activation inhibition through PI3K/Akt pathway.

In summary, our current study elucidated that wogonoside could prevent colitis-associated colorectal carcinogenesis and inhibit colon cancer progression in inflammation-related microenvironment via inhibiting NF-κB activation through PI3K/Akt pathway. Therefore, wogonoside might be a promising effective chemotherapeutic agent for inflammation-related cancer in the future.

## MATERIALS AND METHODS

### Reagents and antibodies

Dimethylsulfoxide (DMSO) was purchased from Sigma-Aldrich (St. Louis, MO, USA). Sodium carboxyl methyl cellulose (CMC) was obtained from Sinopharm Group Co. Ltd. (Shanghai, China). Wogonoside (>98% purity; Langze Pharmaceutical Co, Ltd, Nanjing, China) was dissolved in dimethylsulfoxide (DMSO) as stock solution at 0.1 M, stored at −20°C, and freshly diluted with RPMI-1640 medium (Gibco, Carlsbad, CA) to the final concentration in *in vitro* study. In *in vivo* study, wogonoside was prepared as intragastric administration (0.5% CMC) by Dr. Xue Ke from College of Pharmacy, China Pharmaceutical University.

LPS (E. coli: Serotype O55:B5), 3-(4,5-dimethylthiazol-2-yl)-2,5-di- phenyltetrazolium bromide (MTT) and Azoxymethane (AOM) were purchased from Sigma-Aldrich (St. Louis, MO, USA). Dextran sulfate sodium (DSS, molecular weight 36-50 kDa) was obtained from MP Biomedicals Inc. (Irvine, CA, USA). Dye DAPI was purchased from Invitrogen (Carlsbad, CA, USA). Paraformaldehyde (PFA) was purchased from Yonghua Chemical Technology (Jiangsu) Co. Ltd. (Changshu, China). Triton X-100 was purchased from Shanghai Chao Rui Biotech. Co. Ltd. (Shanghai, China). Insulin-like growth factor-1 (IGF-1) was obtained from PeproTech (Suzhou, China). LY294002 were from Beyotime (Shanghai, China). BSA was purchased from Roche Diagnosis (Shanghai) Ltd. (Shanghai, China). Agarose was the product from Basingstoke (England). Primary antibodies against Lamin A, IκBα, NF-κB and β-actin were obtained from Santa Cruz Biotechnology (Santa Cruz, CA, USA); PI3K was from Bioworld (Bioworld, OH, USA) and antibodies against Akt, p-Akt, p-IκBα, IKKα, p-IKKα, Cyclin D1 and survivin were purchased from Cell Signaling Technology (Danvers, MA); phospho-p65 was purchased from Epitomics (Burlingame, CA, USA). IRDyeTM800 conjugated secondary antibodies were obtained from Rockland Inc. (Philadelphia, PA, USA). FITC-anti-F4/80 and PE-anti-Gr-1 were purchased from eBioscience (San Diego, CA, USA). Ki67 cell proliferation Detection Kit was from Keygen Biotech (Nanjing, China).

### Cell culture and conditioned culture

HCT116 cells, HT29 cells and THP-1 cells were cultured in RPMI-1640 medium (Gibco, Carlsbad, CA, USA), supplemented with 10% fetal bovine serum (Gibco, Carlsbad, CA, USA), 100 U/ml benzyl penicillin and 100 mg/ml streptomycin. Cells were cultured in a humidified environment with 5% CO_2_ at 37°C. After adding 1 μg/ml LPS into THP-1 cells for 12 h, the medium was removed and cultured with free serum medium for another 12h. Then the supernatant of THP-1 cells was collected by centrifuging as 4000 rpm/min for 10 minutes. HCT116 and HT29 cells were cultured with the supernatant in the absence or presence of wogonoside for 24h. (In the conditional culture system the ratio of THP-1 and HCT116 was 5:1.)

### Cell viability assay

Cell viability was measured using the colorimetric MTT assay as described previously [47]. Experiments were performed in triplicate in a parallel manner for each concentration of wogonoside used and the results were presented as mean ± SD. After incubation for 24 h, absorbance (A) was measured at 570 nm using a Universal Microplate Reader (EL800, BIO-TEK Instruments Inc.). Survival ratio (%) was calculated using the following equation: survival ratio (%) = (Atreatment/Acontrol) × 100 where Atreated and Acontrol are the average absorbance of three parallel experiments from treated and control groups, respectively. IC50 was taken as the concentration that caused 50% inhibition of cell viabilities and calculated by the Logit method.

### Cell proliferation detection

To detect cell proliferation, cells were harvested after treatment and then processed with Ki67 cell proliferation Detection Kit according to the manufacturer's instructions. Observation was taken under a light microscope.

### Soft agar colony-formation assay

The experiment was carried out after HCT116 and HT29 cells were treated with LPS-activated THP-1 conditioned medium with or without wogonoside for 24 h. Cells were seeded in 6-well plates at 10 000 cells/well in 0.8% agar in RPMI-1640 culture medium over a 1.2% agar layer. Plates were further incubated for 28 days until the colonies were large enough to be visualized. The colonies were pictured at 40 magnification to detect colony size and colony numbers, using an inverted microscope equipped with a color camera (Nikon Instruments, Inc., Lewisville, TX).

### AOM/DSS-induced colitis-associated colorectal carcinogenesis and design of drug treatment

6-8 weeks old C57BL/6 mice, weighing 18-22 g, were supplied by Shanghai Laboratory Animal Center, China Academy of Sciences. Experimental protocols were in accordance with National Institutes of Health regulations and approved by the Institutional Animal Care and Use Committee. Throughout the acclimatization and study periods, all animals had access to food and water *ad libitum* and were maintained on a 12 h light/dark cycle (21±2°C with a relative humidity of 45±10%).

AOM/DSS model was established to induce colitis-associated colon cancer (CAC). Briefly, mice were injected intraperitoneally (i.p.) with 12.5 mg/kg AOM and maintained on a regular diet and water for 7 days. After 7 days, mice received 2.5% DSS in drinking water for 7 days. After this, mice were maintained on regular water for 14 days and subjected to two more DSS treatment cycles. The mice were given wogonoside 100 mg/kg every day via gastric intubation starting 7 days before the AOM injection, until the termination of the experiment. Body weight was measured every week. On day 106, mice were killed. Macroscopic tumors were counted and measured with a caliper. The clinical course of the disease was followed daily by measuring body weight and monitoring for signs of rectal bleeding or diarrhea.

### Macroscopic assessment and histological analysis of colonic lesions

After AOM/DSS-induced CAC, the animals were sacrificed and colons were removed, opened longitudinally, and washed with phosphate-buffered saline (PBS). The pieces of colonic tissue were used for *ex vivo* analysis. The histological analysis was performed as previously described [48].

### Immunofluorescence (IF) of colon tissues

The neutrophil (Gr-1^+^) and macrophage (F4/80^+^) infiltration analysis was performed on paraffin-embedded colon tissue sections. Briefly, the sections were deparaffinized, rehydrated and washed in 1% PBS Tween. Then they were treated with 3% hydrogen peroxide, blocked with 5% bovine serum albumin (BSA) and incubated for 1 h at room temperature with anti-F4/80^+^ or anti-Gr-1^+^ (1:100). The slides were then counter-stained with DAPI for 30 min. The reaction was stopped by thorough washing in water for 5 min. Images were acquired by confocal laser-scanning microscope (Olympus, Lake Success, NY). Settings for image acquisition were identical for control and experimental tissues.

### Immunohistochemistry (IHC)

The expression of Ki67, PCNA, BrdU, NF-κB, IL-1β, TNF-α and IL-6 of the colonic tissues was assessed as described in the previous study [49].

### Preparation of cytosolic and nuclear extracts and whole cell lysates

Nuclear and cytosolic protein extracts were prepared according to the modified method as described previously [50]. The cytosolic and nuclear fractions was subjected to immunoblot analysis. The whole cell lysates were prepared as described previously [51].

### Western blot analysis

The cytosolic, nuclear extracts and whole cell lysates were prepared and subjected to Western blot analysis as described previously [50]. Bands were detected immunologically using antibodies against PI3K (1:800), Akt (1:800), p-Akt (1:800), IKKα (1:800), p-IKKα (1:800), p-IκBα (1:800), Cyclin D1 (1:800), survivin (1:800) NF-κB (1:500), phospho-p65 (1:500). All blots were stripped and reprobed with β-actin (1:2000) or Lamin A (1:2000) antibody to ascertain equal loading of proteins.

### Immunofluorescence confocal microscopy

The immunofluorescence assay for NF-κB nuclear translocation was performed according to the method previously described [50]. Then cells were immunostained with Alexa Fluor 488-conjugated anti-rabbit IgG (Life technology, CA) for 2 h. The images were captured with an Olympus FV1000 confocal microscope.

### Luciferase assay

Luciferase activities were measured as previously described [52]. Luciferase activities were expressed as fold induction relative to values obtained from control. The results represent the mean of at least three independent experiments, each carried out in duplicate. The GFP luciferase activity was used as an internal control for transfection efficiency.

### Quantitative real-time PCR analysis

Total RNA isolation and real-time PCR were performed as previously described. The primers in the reaction were used as follows:

Mouse IL-6-sense (5′-ACAACCACGGCCTTCCC TAC-3′);
Mouse IL-6-antisense (5′-TCTCATTTCCACGATTTCCCAG-3′);
Mouse IL-1β-sense (5′-CCAAGCTTCCTTGTGCAAGTA-3′);
Mouse IL-1β-antisense (5′-AAGCCCAAAGTCCAT CAGTGG-3′);
Mouse β-actin-sense (5′-TGCTGTCCCTGTATGCC TCT-3′);
Mouse β-actin-antisense (5′-TTTGATGTCACGCACGCACGATTT-3′);
Mouse TNF-α-sense (5′-CGAGTGACAAGCCTGT AGCCC-3′);
Mouse TNF-α-antisense (5′-GTCTTTGAGATCCATGCCGTTG-3′).

### Statistical analysis

The data shown in the study were expressed as means ± SD from at least three independent experiments, each in triplicate samples for individual treatment or dosage. Statistical analyses were performed using ANOVA coupled with a post hoc test.

## Abbreviations

AOM: azoxymethane
DSS: dextran sulfate sodium
CRC: colorectal cancer
IBD: inflammatory bowel disease
CAC: colitis-associated colon cancer
NF-κB: nuclear factor kappa B
PI3K: phosphatidylinositol-3-kinase
IKK: I-κB kinase
PCNA: proliferating cell nuclear antigen
MTT: 3-(4,5-dimethylthiazol-2-yl)-2,5-di-phenyltetrazolium bromide
LPS: lipopolysaccharides
BSA: bovine serum albumin
DAPI: diamidino-phenyl-indole
DMSO: dimethylsulfoxide
TUNEL: TdT-mediated dUTP-biotin nick end labeling
BrdU: 5-Bromo-2-deoxyUridine
IL-1β: interleukin-1 beta
IL-6: interleukin-6
PBS: phosphate-buffered saline
TNF-α: tumor necrosis factor alpha.
